# A physical activity program versus usual care in the management of quality of life for pre-frail older adults with chronic pain: randomized controlled trial

**DOI:** 10.1186/s12877-020-01805-3

**Published:** 2020-10-08

**Authors:** Pedro Otones, Eva García, Teresa Sanz, Azucena Pedraz

**Affiliations:** 1 San Andrés Primary Care Center, Gerencia Asistencial de Atención Primaria, Alberto Palacios, 22, 28021 Madrid, Spain; 2 Nursing Department, Universidad Autónoma de Madrid, Madrid, Spain; 3 Research Unit, Gerencia Asistencial de Atención Primaria, Madrid, Spain; 4 Health Services Research on Chronic Patients Network (REDISSEC), Instituto Salud Carlos III, Madrid, Spain

**Keywords:** Frailty, Chronic pain, Exercise, Quality of life, Aged, Nursing education

## Abstract

**Background:**

Exercise has shown being effective for managing chronic pain and preventing frailty status in older adults but the effect of an exercise program in the quality of life of pre-frail older adults with chronic pain remains unclear. Our objective was to evaluate the effectiveness of multicomponent structured physical exercise program for pre-frail adults aged 65 years or more with chronic pain to improve their perceived health related quality of life, compared with usual care.

**Methods:**

Open label randomized controlled trial. Participants were community-dwelling pre-frail older adults aged 65 years or older with chronic pain and non-dependent for basic activities of daily living attending a Primary Healthcare Centre. Forty-four participants were randomly allocated to a control group (*n* = 20) that received usual care or an intervention group (*n* = 24) that received an 8-week physical activity and education program. Frailty status (SHARE Frailty Index), quality of life (EuroQol-5D-5L), pain intensity (Visual Analogue Scale), physical performance (Short Physical Performance Battery) and depression (Yessavage) were assessed at baseline, after the intervention and after 3 months follow-up. The effect of the intervention was analysed by mean differences between the intervention and control groups.

**Results:**

The follow-up period (3 months) was completed by 32 patients (73%), 17 in the control group and 15 in the intervention group. Most participants were women (78.1%) with a mean age (standard deviation) of 77.2 (5.9) years and a mean pain intensity of 48.1 (24.4) mm. No relevant differences were found between groups at baseline. After the intervention, mean differences in the EuroQol Index Value between control and intervention groups were significant (− 0.19 95% CI(− 0.33- -0.04)) and remained after 3 months follow-up (− 0.21 95% CI(− 0.37- -0.05)). Participants in the exercise group showed better results in pain intensity and frailty after the intervention, and an improvement in physical performance after the intervention and after 3 months.

**Conclusions:**

An eight-week physical activity and education program for pre-frail older adults with chronic pain, compared with usual care, could be effective to improve quality of life after the intervention and after three-months follow-up.

**Study registration details:**

This study was retrospectively registered in ClinicalTrials.gov with the identifier NCT04045535.

## Background

Chronic pain (CP) is one of the most frequent, costly and incapacitating conditions in older adults [1]. It is usually accompanied in older adults with atypical symptoms, comorbidities, polypharmacy and increased risk of interactions and secondary effects, which makes its management complex [2]. Important age modifications in pain perception has been described with a reduction in the descending inhibitory capacity and an increase in pain alert thresholds due to an impairment of Aδ fibres and a decline in concentration of catecholamines, GABA and opioid receptors [2, 3]. Those differences make pain assessment and treatment complex. The failure to effectively identify and manage pain could result in a reduced quality of life and could impact negatively on the relationship between the older person and the caregiver [4].

Chronic Pain is a highly prevalent condition. The last Global Burden of Disease identified among the commonest chronic conditions (over 1% of global prevalence) several primary pain conditions: migraines, low back pain, neck pain, musculoskeletal conditions or osteoarthritis [5]. The prevalence of CP in Europe among older adults according to The Survey of Health, Ageing and Retirement in Europe (SHARE) was 35.7% (95% CI 34.9–36.5) and this prevalence was higher in women (41.3%; 95% CI 40.2–42.4) [6]. Chronic Pain conditions were associated with frailty, poor mobility, depression, cognitive impairment, falls, and low physical activity (PA) levels [7].

The World Health Organization (WHO) recommends that all people aged 65 years or above should do 150 min or more of moderate-intensity physical activity per week or 75 min of higher intensity PA [8]. Nevertheless, exercise participation declines progressively through adult life [9]. Physical inactivity is after high blood pressure, tobacco consumption and high blood glucose concentrations, the fourth most important risk of mortality worldwide [10]. It has been shown that regular PA participation reduces the risk of coronary heart disease, diabetes, hypertension, stroke, some types of cancer and depression [8]. It can also increase years living independently, reduce disability, improve quality of life and reduce mortality for all causes [11]. People who participate in physical exercise programs are less frequently classified as frail [12].

Frailty is a common and potentially incapacitating condition in older adults. It is defined as a clinical state in which an individual’s vulnerability for developing dependency and/or mortality when exposed to a stressor increases [13]. It is associated with older age, female sex, lower incomes or polypharmacy [14]. Frailty should be assessed by validated tools, that usually characterize patients in three types, according to their functional status: frail, pre-frail or robust [13]. A strong association between a frail or pre-frail status and lower quality of life has been reported by several investigations [15]. The prevalence of diabetes, heart disease, osteoporosis, lung disease and stroke is twice that in robust, and the prevalence of having suffered falls in the previous year is three to four times higher [16]. Falls are one of the major consequences of frailty. They are the second leading cause of accidental deaths worldwide [17] and more than 70% of falls have clinic consequences like fractures or injuries [18]. Older women with lower levels of physical activity report higher risk of falls than women that usually participate in physical activities [19].

An association between suffering from CP and being classified as frail or pre-frail has been reported in several investigations [20, 21]. It has also been reported that adults with CP participate in less PA than individuals with no pain [22]. Nevertheless, it has been reported that physical activity performance may change pain modulation in older adults [23]. Due to the impact in quality of life of frailty, CP and lower PA and the potential benefits of PA programs for frailty prevention and CP management our aim is to evaluate the effectiveness of a multicomponent structured physical exercise program for adults aged 65 years or more, classified as pre-frail and with chronic pain to improve their perceived health related quality of life, compared with usual care in primary health care.

## Methods

### Design

This study was designed as a parallel group open label randomized controlled trial with repeated measures and 3 months follow-up to determine the effect of an eight-week physical activity program for pre-frail older adults with chronic pain. This study adheres to the CONSORT Statement for Non-pharmacologic Treatment Interventions [24].

### Participants and setting

Data were collected between May 2018 and June 2019 in a Primary Health Care Centre in Madrid, Spain. All participants classified as pre-frail (*n* = 59; 47 women and 12 men) in a previous cross-sectional investigation about frailty status in older adults with chronic pain were invited to participate in this randomized controlled trial. They were included if they accepted to participate and met the inclusion criteria. Participants were included if they were older adults aged 65 years or older with chronic pain for more than 3 months and classified as pre-frail by the SHARE Frailty Index [25]. Participants were excluded if they were dependent for activities of daily living (ADL). Dependence for ADL has been measured with the Barthel ADL index [26, 27]; participants with index result under 90 were excluded. They were also excluded if they were classified as a homebound person. It is defined as persons who never or rarely left the home in the past month [28]. Participants unable to answer questionnaires independently, due to mental illness, dementia or language barriers were also excluded. Participants were excluded if they were institutionalized, if they lived out of the area of the investigation for more than 6 months per year or if they were participating in other clinical trials. Simple randomization was developed using the program Epidat 4.2 to randomly assign participants after signing the written consent to an intervention group or control group. Nor participants or researchers knew the allocation group when they were invited to participate and signed the written consent.

### Control group

Participants in the control group received usual practice in Primary Care, which included an assessment of dependence with Barthel Index, frailty assessment with Short Physical Performance Battery and structured education about nutrition, physical exercise, moderate sun exposure, falls prevention and medication use. They were invited to participate in the physical activity program after the trial was finished if results were positive and they accepted.

### Intervention group

Participants in the intervention group received an eight-week physical activity and education program in addition to usual practice in Primary Care. The physical activity and education program was adapted from a similar program developed by Tse et al. (2014) for older persons living in nursing homes that reduced pain intensity and improved emotional wellness of the participants reporting better results in happiness, loneliness, life satisfaction and depression [29].

The physical activity and education program was developed once a week for 8 weeks in a conference and multi-function room in the Primary Care Centre. Each session lasted for 60 min, 15 min for warming-up and 45 min for exercises that changed in each session: shoulder and neck exercises; back exercises; knee and ankle exercises; hip exercises; balance exercises; falls prevention education; questions, answers and reflections; revision, reflections, evaluation and goodbye. Participants made suggested exercises accompanied by the instructor. After each session, they received a document with pictures that described the exercises of the day.

### Measures

The main outcome variable in this study was perceived health-related quality of life. Data on pain intensity, frailty status, physical performance, depression, basic activities of daily living and satisfaction with the intervention were also recorded.

#### Quality of life

Participants’ perceived health related quality of life was assessed with the EuroQol 5D-5L (EQ-5D-5L) [30]. This questionnaire consists of five dimensions of health (mobility, self-care, usual activities, pain/discomfort and anxiety/depression) with five levels of problems. It also includes a Visual Analogue Scale with a range 0–100 in which higher scores indicates better quality of life. The results of the five dimensions are transformed in an index value by a calculator with different value sets depending on the setting country which has been validated in our context [31].

#### Pain intensity

It was assessed with the Visual Analogue Scale of the Short Form of McGill Pain Questionnaire [32] ranging from “no pain= 0mm” to “unwilling pain= 100mm”. Scores were calculated to the nearest millimetre with a ruler. Minimally clinically important difference for VAS is 23 mm [33].

#### Frailty

Subjects were classified as frail, pre-frail or robust according to the five criteria of the Survey of Health, Ageing and Retirement in Europe Frailty Index (SHARE-FI) [25]:

Exhaustion: by the positive response to the question: ‘In the last month, have you had too little energy to do the things you wanted to do?’

Weight loss: by reporting a diminution in desire for food in response to the question: ‘What has your appetite been like?’

Weakness: assessed by handgrip strength using a dynamometer twice in each hand. The highest measurement is selected.

Slowness: by positive answer to one of the following questions: ‘Because of a health problem, do you have difficulty walking 100 meters or climbing one flight of stairs without resting?’

Low activity: assessed by the question: ‘How often do you engage in activities that require a low or moderate level of energy such as gardening, cleaning the car or doing a walk?’

The aim of the SHARE-FI is to summarize those variables in a single discreet factor (DFactor) with three classes: non-frail, pre-frail and frail. Changes in 0.5 DFactor score (DFS) were described as an improvement in frailty status [34]. The formula for the DFS is different in men and women:

Women: If predicted DFS < 0.31, NON-FRAIL; if predicted DFS < 2.13, PRE-FRAIL; if predicted DFS < 6, FRAIL.

Men: If predicted DFS < 1.21, NON-FRAIL; if predicted DFS < 3.00, PRE-FRAIL; if predicted DFS < 7, FRAIL.

#### Physical performance

The Short Physical Performance Battery (SPPB) was used [35]. It consists of three tests: balance skills, gait speed and chair stands. Balance is assessed using foots side-by-side, semi-tandem and tandem stands. Gait speed was tested with two four meters walk with or without mobility devices. The ability to stand from a chair and return to seated position five times with arms crossed was also tested. A final score was calculated ranging from zero (worst performance) to twelve (best performance).

#### Basic activities of daily living

Basic Activities of Daily Living (bADL) dependence was assessed by Barthel ADL index [26, 27]. It assesses the help needed with ten variables: feeding, bathing, grooming, dressing, urinary incontinence, faecal incontinence, toilet use, transfers bed to chair, mobility and climbing stairs.

#### Depression

Depression was assessed using the 5-items Geriatric Depression Scale [36]. It comprises five questions with yes or no answers. It is a commonly used tool in Primary Care and it has been validated in our context [37].

#### Further measurements

Satisfaction with the program was assessed using the 8-items Clients Satisfaction Questionnaire (CSQ-8) [38]. It is a generic questionnaire with 8 questions (4 of them with reverse score) about participants’ satisfaction with the intervention received, if they feel that the intervention was useful and if they would recommend it to their counterparts.

Sociodemographic characteristics like age, sex or if participants live alone or in family were also recorded.

### Statistical analysis

Data were recorded in an electronic database for analysis. Qualitative and categorical variables were described using numbers of participants (N) and percentages, and continuous variables were described using means and standard deviations (SDs). Chi-square test was used to analyse the differences in the qualitative data between the intervention and control group. T-test analyses were used to compare the differences of the quantitative outcomes’ variables. We calculated the difference between the means in each group, post-intervention time and at 3-month follow-up with their 95% CI. All differences were assessed for their clinical significance as shown in previous studies. All analyses were performed using IBM SPSS Statistics v.23.

### Ethics

All participants agreed to participate in the study by signing a written informed consent. The protocol of the investigation was registered in the database of clinical studies ClinicalTrials.gov (ClinicalTrials.gov Identifier: NCT04045535) and it was approved by two independent ethical research committees from a hospital (Hospital 12 de Octubre, code: 18/170) and a university (Universidad Autónoma de Madrid, code: CEI-88-1659).

## Results

### Participants’ demographic and clinical characteristics

**Fig. 1:**
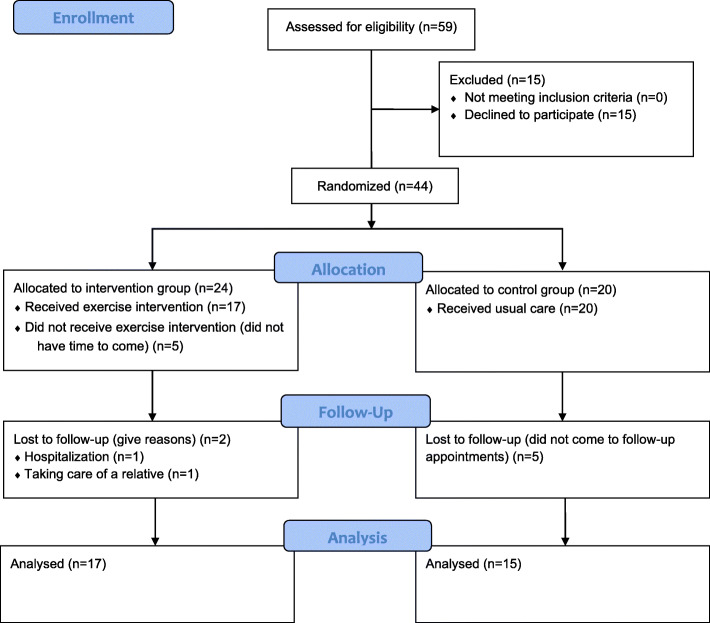
CONSORT flow diagram

Fifty-nine pre-frail older adults with chronic pain were invited to participate in the study. Fifteen patients refused to participate and 44 were randomly assigned to the control or intervention group. Finally, 32 participants finished the randomized controlled trial and were analysed, this was the result of a loss of participants in both groups during the 3 months follow-up due to not being able to come to the intervention (intervention group, *n* = 5), not coming to follow-up (control group, *n =* 5), hospitalisation (intervention group, *n* = 1) and illness of a relative (intervention group, *n =* 1) (Fig. 1).

Participants had a mean (Standard Deviation, SD) age of 77.2 (5.9) years and a mean (SD) pain intensity of 48.1 (24.4) mm. There were a majority of women, 78.1 and 21.9% were men. No statistically significant differences were found between control group and intervention group excluding mean age of intervention group participants, which were 4.9 years older than control group participants (Table 1).

**Table 1:** Baseline demographic and clinical characteristics by group

		Total	Intervention	Control	***p***
		(***n*** = 32)	(***n*** = 17)	(***n*** = 15)	
**Sex**	**n (%)**				
**Male**		7 (21.9%)	5 (29.4%)	2 (13.3%)	.27
**Female**		25 (78.1%)	12 (70.6%)	13 (86.7%)	
**Age**	**Mean (SD)**	77.2 (5.9)	79.5 (4.2)	74.6 (6.5)	.02
**Pain VAS**	**Mean (SD)**	48.1 (24.4)	41.9 (18.3)	55.2 (28.9)	.13
	**Median (IQR)**	50.5 (35–69.75)	40 (29.5–57.5)	68 (39–73)	
**SPPB**	**Mean (SD)**	7.1 (1.9)	6.6 (1.7)	7.7 (1.9)	.11
**Quality of life**					
**EQ-5D VAS**	**Mean (SD)**	60.9 (18.7)	60.5 (17.7)	61.3 (20.4)	.91
	**Median (IQR)**	60 (50–73.5)	60 (50–65)	60 (50–80)	
**Index value**	**Mean (SD)**	.61 (.22)	.67 (.13)	.55 (.28)	.12
	**Median (IQR)**	0.69 (0.51–0.75)	0.7 (0.56–0.76)	0.69 (0.46–0.74)	
**Mobility**	**n (%)**	11 (34.4%)	4 (23.5%)	7 (46.7%)	.11
**Self-care**	**n (%)**	4 (12.5%)	1 (5.9%)	3 (20%)	.86
**Usual activ.**	**n (%)**	12 (37.5%)	6 (35.3%)	6 (40%)	.45
**Pain**	**n (%)**	22 (68.8%)	12 (70.6%)	10 (66.7%)	.88
**Anxiety**	**n (%)**	12 (37.5%)	6 (35.3%)	6 (40%)	.31
**Yesavage**	**Mean (SD)**	1.5 (1.2)	1.4 (1.2)	1.8 (1.2)	.39
**Barthel**	**Mean (SD)**	97.6 (3.1)	97.6 (3.1)	97.6 (3.2)	.99
**Number of medications**	**Mean (SD)**	9.2 (3.8)	9.3 (4.4)	9.2 (3.1)	.92
**Live with**	**n (%)**				
**Family**		4 (12.5%)	3 (17.6%)	1 (6.7%)	
**Partner**		17 (53.1%)	8 (47.1%)	9 (60.0%)	.60
**Alone**		11 (34.4%)	6 (35.3%)	5 (33.3%)	

*VAS* Visual Analogue Scale, *SD* Standard Deviation, *IQR* Inter-quartile range, *SPPB* Short Physical performance battery

### Primary outcomes

As shown in Table 1, no baseline (T0) clinical differences were found between intervention and control groups in quality of life index value, VAS, or percentage of moderately, severely, or extremely affected EQ-5D-5L dimensions. At T1 both groups improved their results in the EQ-5D-5L index value. The mean score (SD) of the intervention group (0.78 (0.12)) was significantly higher than the score of the control group (0.59 (0.25)). After the 3 months follow-up (T2), differences between results of the intervention group (0.77 (0.13)) and the control group (0.56 (0.28)) remained being statistically significant (Table 2).

**Table 2:** Mean differences between intervention and control groups

	Post-intervention (T1)			3 months follow-up (T2)		
	Mean differences	95% CI	***p***	Mean differences	95% CI	***p***
**Quality of life**						
**Index value**	−0.19	(−0.33- -0.04)	**.02**	−0.21	(−0.37- -0.05)	**.01**
**EQ-5D VAS**	−9.1	(−23.58–5.39)	.21	−4.3	(−15.22–6.63)	.43
**Frailty**						
**DFS Men**	−1.19	(−3.46–1.09)	.24	0.1	(−2.16–2.36)	.91
**DFS Women**	−1.22	(−1.96- -0.48)	**.00**	−0.82	(−1.54- -0.10)	**.03**
**VAS pain**	22.12	(4.18–40.06)	**.02**	13.49	(−4.09–31.07)	.13
**SPPB**	0.29	(−1.17–1.15)	.69	0.39	(−1.23–2.00)	.62
**Yesavage**	−0.12	(−1.18–0.93)	.82	−0.28	(−1.13–0.58)	.51
**Barthel**	−0.27	(−2.55–2.00)	.81	−1.65	(−5.06–1.76)	.33

*VAS* Visual Analogue Scale, *CI* Confidence Interval, *SPPB* Short Physical performance battery, *DFS* Discreet factor score

### Pain intensity measures for the intervention and control groups

After finishing the physical activity program (T1), differences in mean (SD) pain intensity were found between the intervention group (29.88 (14.94)) and the control group (52.00 (30.19)), as shown in Table 2. At T2, differences did not remain being statistically significant, mean (SD) pain intensity for the intervention group (38.18 (20.65)) and for the control group (51.67 (27.90)) was comparable to baseline measures.

### Physical performance measures for the intervention and control groups

After the completion of the physical activity program (T1), compared to baseline, the mean (SD) result of the SPPB was significantly higher for the intervention group (8.18 (1.78), *p* = .01). These results remained after 3 months follow-up (8.41 (1.77), *p* < .01). Control group also improved the results of the SPPB at T1 (8.47 (2.26), *p* = .06) and T2 (8.8 (2.54), *p* = .03) but not as significantly as the intervention group. No statistically significant differences were found between groups (Table 2).

### Frailty status for the intervention and control groups

At T0, all participants were classified as pre-frail. After the intervention (T1), 58.8% of the participants in the intervention group were classified as non-frail and 41.2% as pre-frail. In the control group 80% of participants remained as pre-frail at T1, 13.3% were classified as frail and 6.7% as non-frail. At T2, most participants of both groups (70.6% intervention group and 73.3% in control group) were classified as pre-frail but there were more participants classified as non-frail in the intervention group than in the control group (23.5% vs 13.3%).

#### Further measures

No differences were found between the intervention and control groups in depression or bADL, as shown in Table 2.

Participants reported a high satisfaction with the intervention with a mean satisfaction of 30.69/32 points. No side effects were reported by the participants during the physical activity education program or during the three-months follow-up.

## Discussion

In this randomized controlled trial, we found that compared to the control group, participants who received a physical activity intervention reported better quality of life, better frailty status and lower pain intensity. Furthermore, participants in the intervention group reported better physical performance after the intervention and after 3 months follow-up than at baseline. These results are consistent with those of other studies on older adults. The effect of physical activities in reversing frailty status, reducing the fear of falls or improving mobility have been reported by previous systematic reviews [39]. Exercise and nutrition interventions showed being more effective to reverse frailty status in community-dwelling frail women than only nutritional interventions [40].

Our trial found an improvement in quality of life assessed with EQ-5D-5L in the intervention group of 0.10 points at T1 and 0.09 points at T2 from baseline. Previous investigations found that a change of 0.03 points was related to a change on the global perceived effect of a treatment in patients with chronic low-back pain [41] and that minimal clinically important difference (MCID) for improvement was 0.07 points in patients with osteoarthritis [42]. Previous studies showed an association between quality of life and physical performance, patients with lower scores in the SPPB also reported worse results in the EQ-5D-5L index value [43]. Reporting increased pain sites or pain severity was also associated with poorer SPPB results [44]. Our research found an improvement in the physical performance assessed with the SPPB in the group that received the exercise intervention. The improvement was higher than these identified as substantial changes in previous investigations [45].

Although no differences in depression assessed with the 5-Item Geriatric Depression Scale were found, we found statistically significant differences between control and intervention group in the anxiety/depression dimension of EQ-5D-5L. These results are consistent with those of the investigation of Tse et al. (2014) [29]. They found significant changes in happiness, loneliness, life satisfaction and depression in the exercise group. A recent qualitative research revealed that older adults participating in a community-based program of socialization, health education exercise and walking reduced their feelings of loneliness and social isolation [46].

A recent overview of 21 Cochrane Reviews with 381 included studies found that, although further research is required, exercise interventions produce small-to-moderate positive effect in pain severity and physical function and consequently quality of life, with few adverse events and some effects in psychological function [47]. In our research, participants in the intervention group showed a reduction of 12.06 mm of the VAS at T1, but that reduction in pain intensity was not maintained after the three-months follow-up. Although there were differences in pain intensity in the intervention group between T0 and T1 and those differences were statistically significant, the small effect of the intervention in pain intensity made that clinical relevance could be questionable.

Despite the positive effects of the physical activity program, this study has several limitations. First, the number of participants is limited, which makes it difficult to generalize the results of the intervention. They were recruited from a previous observational investigation about prevalence of frailty in patients with chronic pain and it could not include all pre-frail older adults with chronic pain of the study area. Moreover, there is no description of painkillers use and the location, cause, and period of chronic pain. Second, the investigation was developed just in one Primary Health Care Center, which could affect the external validity of the results. Third, due to the characteristics of the intervention, it was impossible to blind participants or researchers. When participants were proposed to participate in the investigation nor them or the researcher knew the group they were going to be assigned to.

Future research could be necessary to solve the limitations that were found in this trial, with a larger number of participants, differentiating period, grade, and area of chronic pain, and including not only pre-frail participants but also frail and robust ones. A multicenter research could include participants with different socioeconomic characteristics and living in urban or rural areas which could be interesting to evaluate how physical activity affects older adults with chronic pain in different environments.

## Conclusion

An eight-week physical activity and education program for pre-frail older adults with chronic pain, compared with usual care, could be effective to improve quality of life after the intervention and after three-months follow-up. Participants who received the physical activity and education program also showed better physical performance and reported high satisfaction with the intervention.

## Data Availability

The datasets generated and analyzed during the current study are not publicly available due to not having consent from the patients to disclose raw data.

## Abbreviations

ADL: Activities of daily living
bADL: Basic Activities of Daily Living
CI: Confidence Interval
CONSORT: Consolidated Standards of Reporting Trials
CP: Chronic Pain
CSQ-8: Client Satisfaction Questionnaire 8 Items
DFS: Discreet factor Score
EQ-5D-5L: EuroQol 5 dimensions and 5 levels.
GABA: Gamma-Aminobutyric acid.
IQR: Inter-quartile range.
MCID: Minimal clinically important difference.
PA: Physical Activity.
SD: Standard Deviation
SHARE: Survey of Health, Ageing and Retirement in Europe
SHARE-FI: Survey of Health, Ageing and Retirement in Europe Frailty Index
SPPB: Short Physical Performance Battery
SPSS: Statistical Package for Social Sciences
VAS: Visual Analogue Scale
WHO: World Health Organization
